# 3D passive stabilization of *n* = 0 MHD modes in EAST tokamak

**DOI:** 10.1038/srep32440

**Published:** 2016-09-06

**Authors:** S. L. Chen, F. Villone, B. J. Xiao, L. Barbato, Z. P. Luo, L. Liu, S. Mastrostefano, Z. Xing

**Affiliations:** 1 Institute of Plasma Physics, Chinese Academy of Sciences, Hefei, People’s Republic of China; 2 DIEI, Universitá degli Studi di Cassino e del Lazio Meridionale, Italy; 3 School of Nuclear Science and Technology, University of Science and Technology of China, Hefei, China

## Abstract

Evidence is shown of the capability of non-axisymmetrical conducting structures in the Experimental Advanced Superconducting Tokamak (EAST) to guarantee the passive stabilization of the *n* = 0 MHD unstable mode. Suitable numerical modeling of the experiments allows a clear interpretation of the phenomenon. This demonstration and the availability of computational tools able to describe the effect of 3D conductors will have a huge impact on the design of future fusion devices, in which the conducting structures closest to plasma will be highly segmented.

Tokamaks are toroidal fusion devices in which the plasma evolution can be conveniently described, in many cases, by Magneto-Hydro-Dynamics (MHD) equations^1^. These predict the possible existence of unstable modes of evolution, which can be classified in terms of the toroidal mode number *n* (corresponding to the index of the dominant Fourier harmonic), and are characterized by a growth rate (the rate at which the instability grows in time). In particular, tokamak plasmas with elongated poloidal cross-sections show an intrinsic axisymmetric (*n* = 0) vertical instability^2^, which natively occurs on the very fast Alfvén time scale (typically microseconds), related to the small plasma mass. If a conducting structure is present sufficiently close to the plasma, eddy currents are induced, which tend to counteract the instability itself, giving rise to a passive stabilizing effect lasting until the eddy currents decay, due to non-vanishing resistivity. This intuitively explains why such instabilities are slowed down to electromagnetic timescales (typically milliseconds)^2^, allowing a possible magnetic active feedback control.

In current tokamaks the role of main passive stabilizer is played by the so-called vacuum vessel, which is a toroidally continuous conducting structure in which vacuum is created before the gas is injected and ionized. Consequently, the deviations from axisymmetry of the vessel (e.g. ports) and the other toroidally segmented conducting structures (e.g. blankets) may have an even significant quantitative effect on stabilization^3^, but do not change the qualitative picture of the phenomenon. The EAST tokamak^4^ is a remarkable exception in this respect. Indeed, with reference to the old geometry of EAST (significantly upgraded in 2014) it has been demonstrated^5^ that in this device the vessel is too far from the plasma to provide passive stabilization, which is hence guaranteed by plasma facing components. These are intrinsically non-axisymmetric, being toroidally segmented and connected to the vessel through supports. The aim of this paper is to prove that this happens even with the new geometry of the conductors of EAST, providing a deep physical insight in the phenomenon to support this conclusion. To this purpose, a suitable computational tool will be used, called CarMa0^6^, which has the unique feature of including the effect of 3D conductors on the evolution of fusion plasmas. A careful analysis of the stabilizing current density patterns in the structures and the derivation of an equivalent axisymmetric model allows a clear interpretation and a precise quantification of this effect.

The significance of the results presented in this paper goes beyond the prediction of the behaviour of the EAST tokamak. Indeed, future devices like DEMO^7^ and future fusion power plants will have unavoidably the vessel very far from the plasma, due to the intrinsic need of very thick blankets to slow down fusion neutrons and to provide tritium breeding^7^. Moreover, these blankets will be necessarily toroidally segmented, to allow mounting, replacing and maintenance. Hence, in all these devices, the situation will be most likely very close to what is in EAST today. All the physical understanding gained on EAST, the experimental demonstration of the capability of passive stabilization of toroidal in-vessel components, and the availability of a sound computational tool able to treat this situation, will give a fundamental contribution to the design of these future devices.

## The EAST tokamak

**Figure 1:**
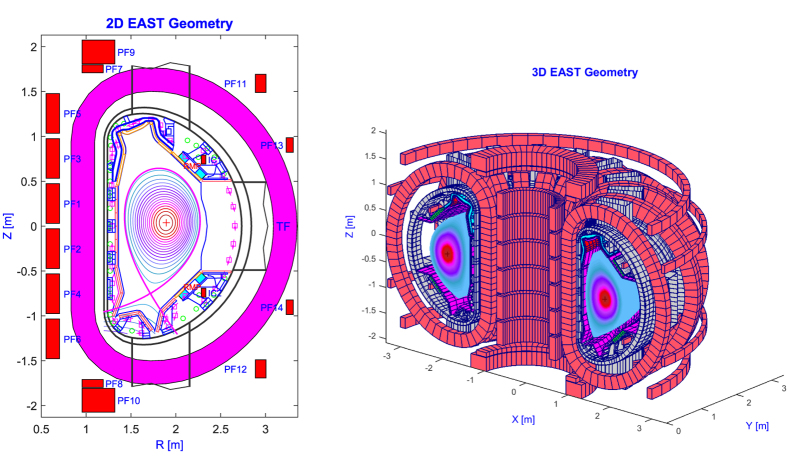
Geometry of the EAST tokamak: 2D view in the poloidal plane and 3D view (one half).

The Experimental Advanced Superconducting Tokamak (EAST)^4^ is an experimental device with fully superconducting poloidal and toroidal coils, designed and constructed to explore the physical and engineering issues under steady state operation for support of future fusion reactors. The main parameters are as follows: major radius 1.8 m, minor radius 0.45 m, toroidal magnetic field 3.5 T, plasma current 1 MA. It can be operated in quite flexible plasma shapes (single-null and double null divertor configurations), with elongation spanning the range 1.5–2.0 and triangularity the range 0.3–0.6. The geometry of the tokamak is reported in Fig. 1.

EAST has recently undertaken an extensive upgrade with enhanced heating and current drive capabilities, with a gross power up to 26 MW since the shutdown after the campaign in 2012. The upper divertor is upgraded to W/Cu plasma facing components (PFCs), with ITER-like W monoblocks as the divertor target structures capable of handling high heat fluxes up to 10 *MW*/*m*^2^, the flat-type divertor dome and baffles allowing for lower heat loads of 4–5 *MW*/*m*^2^. A new internal cryopump is also assembled in the upper divertor behind the outer target to improve divertor particle exhaust. The passive plate structures have been partly modified so as to accommodate the installation of in-vessel resonant magnetic field (RMP) coils and updated in-vessel active vertical stabilization coils (IC coils).

The main conducting structures surrounding the plasma are the vacuum vessel and the in-vessel plasma facing components. The vacuum vessel is welded from 16 sectors with a D-shaped cross section, and made of a double shell of stainless steel (8 mm thickness each), with 3 ports in each sector. It has remained unchanged during the latest upgrade.

**Figure 2:**
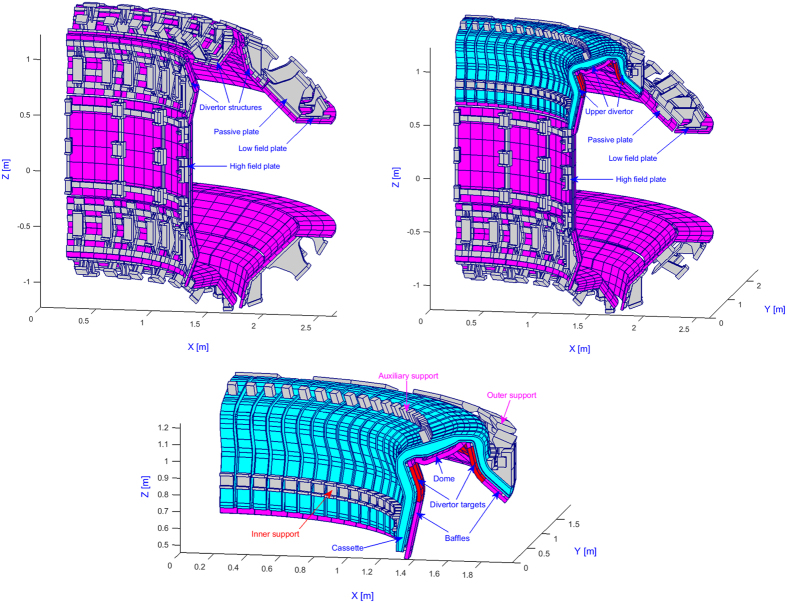
Geometry of the PFCs: old (top left) and new (top right), new upper divertor (bottom).

Conversely, the PFCs have been significantly modified. They consist of low field plates, passive plates, divertor structures (containing outer plates, inner plates and dome) and high field plates (Fig. 2). All these PFCs are toroidally segmented at each sector. As compared to the old geometry, the heat sink of the passive plates has been cut along the toroidal direction (i.e. producing a toroidal gap). Furthermore, the thickness of the heat sink plate is smaller, and simultaneously it is moved closer to the plasma. The support structures of passive plates also have been modified. The upgraded upper divertor is made of divertor targets, a divertor dome, baffles, a cassette structure and support structures. There are in total 80 cassettes (5 per sector) in a circular array held in the location by toroidal continuous rails. The metallic material of the dome and baffles is CuCrZr alloy, while the cassette is made of stainless steel.

## The CarMa0 code

The CarMa0 code^6^ is the *n* = 0 particularization of the CarMa code^8^, successfully applied to several devices and experimentally validated^9^. It is based on the coupling surface approach: a surface *S* is introduced in between the plasma and the conductors. Inside *S*, linearized MHD evolutionary equilibrium equations are solved, assuming the plasma as axisymmetric; neglecting the plasma mass, a response matrix is computed, describing how the plasma reacts (instantaneously) to given external magnetic fields. Numerically, a differential formulation is used and the problem is reformulated in weak form; the resulting equations are solved using second-order triangular finite elements method^10^.

**Figure 3:**
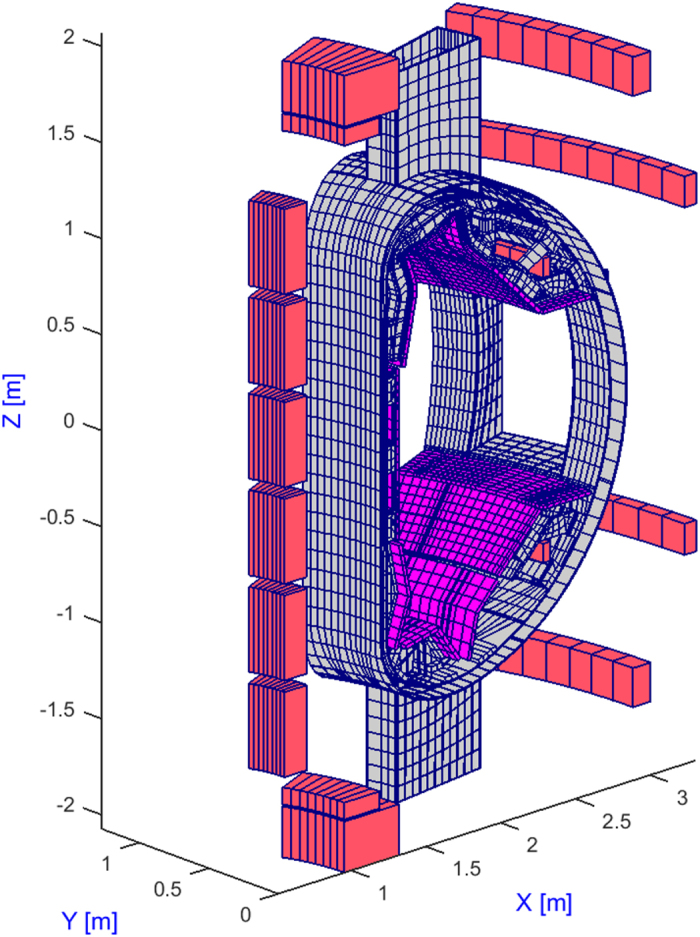
3D finite elements mesh used to describe EAST (one sector).

Conversely, outside *S*, the eddy currents equations are solved inside the conductors, using an integral formulation in terms of the electric vector potential. A 3D volumetric hexahedral finite elements mesh is given of the conductors; edge elements are used as basis functions, to impose the right continuity conditions on current density. Figure 3 reports the finite elements mesh used to represent the EAST tokamak. The mesh features 14907 elements and 27575 nodes; thanks to symmetry, only one sector may be represented.

On the surface *S*, suitable coupling conditions are imposed, by splitting the total magnetic field in terms of the contributions of the plasma current and of the currents induced in external conductors. An equivalent surface current distribution on *S* is computed, able to provide the same magnetic field as plasma outside *S*. Assuming no active coils, the final form of the model^6^ is:


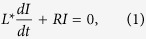


where *I* is a vector of 3D discrete currents in the finite elements mesh, the fully populated inductance matrix L* includes the plasma response in the magnetic interaction among conductors, and the sparse matrix *R* describes the resistance of the 3D structures.

The growth rate *γ* of the *n* = 0 instability is computed as the unstable eigenvalue of the dynamical matrix −(L*)^−1^ *R* of system (1), whose dimensions are of the order of tens of thousands. The corresponding eigenvector can be interpreted as the unstable mode, described in terms of a pattern of 3D currents in the conducting structures.

If the conductors are too far from the plasma, they are not capable of any passive stabilization and the instability is on Alfvén time scale; in the present frame, being the plasma mass neglected, system (1) appears fictitiously stable^2^. The parameter which quantifies when this phenomenon happens is the stability margin^11^, defined as the unstable eigenvalue of the stability matrix −L*L^−1^, where *L* is the inductance matrix of the structures only, without the contribution of the plasma. Indeed, it is possible to demostrate^11^ that when the stability margin goes below 0, then the instability is on Alfvén time scale and the system (1) is fictitiously stable.

## Results

A set of 17 shots has been considered, in which the feedback control of the *n* = 0 instability has been deliberately switched off at a given time instant, giving rise to a Vertical Displacement Event (VDE), in which the vertical position of the plasma column evolves with an exponentially unstable behaviour. The growth rate of the instability can be hence derived experimentally with a suitable linear fit of the logarithm of the experimental trace^12^. The reference equilibrium configurations under analysis feature very different properties, in terms of topology (upper single null, lower single null, double null) and shape. Consequently, the growth rate of the *n* = 0 instability span practically the whole range of interest for EAST, as detailed in the following.

In Table 1 we compare the experimentally measured growth rate *γ* with the modelling results, under several different assumptions. The “3D model” result is the reference computation, using a 3D mesh featuring all the details of the geometry, as described before; evidently, the agreement with experimental data is very good, with a maximum error around 15% and an average error below 2%. Conversely, all the other modelling assumptions provide unsatisfactory results. The “2D model” computation refers to the assumption of purely axisymmetric conducting structures, in particular neglecting the toroidal segmentation of the PFCs and the connections to the vessel; evidently, this assumption provides too low growth rates. The “vessel only” computation considers only a 2D vessel; in most cases, the model is fictitiously stable (N.A. in the table), which means that the vessel alone is not able to provide passive stabilization. The results labelled as “no supports” refer to a fictitious 3D case, in which the connections of the PFCs to the vessel are neglected (this has been approximated imposing a very high resistivity in the supports, hence acting as open circuits). This means that the PFCs are assumed as toroidally segmented, but no current is allowed to flow from the PFCs to the vessel; the growth rates are highly overestimated in this case. The results reported as “equivalent 2D” refer to an equivalent axisymmetric model described in the following.

In Table 2 we report the stability margins computed with various modelling assumptions. Of course, no experimental values are available, since this quantity cannot be directly measured. Evidently, the 2D model is by far too optimistic, as compared to the 3D model.

## Discussion

**Figure 4:**
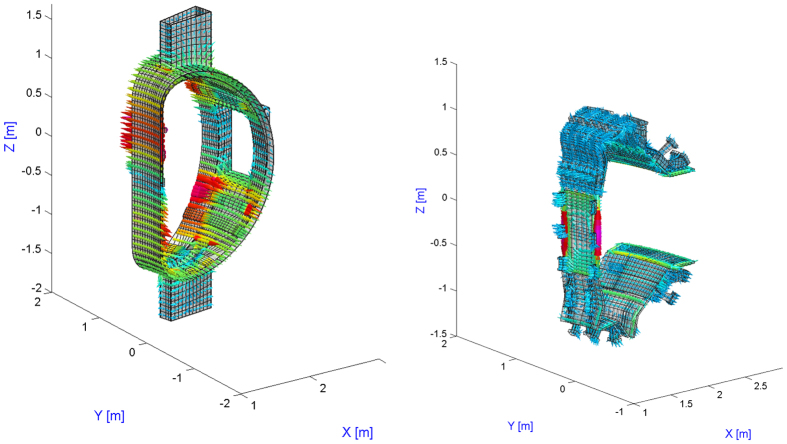
Current density distribution corresponding to the unstable mode.

Now we give a deep physical insight in the results presented above. Figure 4 reports a global view of the current density pattern corresponding to the unstable eigenvector of the dynamical matrix of system (1) for one typical case. The overall current density shows an up-down antisymmetric behaviour, i.e. with currents of opposite directions in the upper and lower parts of the conductors. This pattern is coherent with the reaction to a vertical motion of the plasma, since it mainly produces a radial magnetic field.

**Figure 5:**
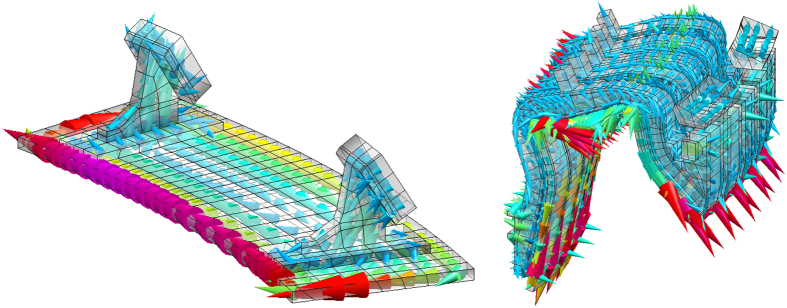
Current density distribution in the low field plate (left) and in the upper divertor (right).

The low field plates have two supports connecting to the vessel inside a sector. The current density flowing in this region is the superposition of a current loop, due to toroidal segmentation, and a “zig-zag” pattern, due to the presence of supports: the current flows from the vessel to the plate through the support, along the plate in the toroidal direction, back to the vessel through the following support. This is clearly shown in Fig. 5. This way, thanks to supports, even with a toroidal segmentation there is a net toroidal current flowing in the plate, which has a significant stabilizing effect, demonstrated by the results of Table 1.

Similarly, in the high field plates, there is a strong current loop, due to segmentation, but also a significant net toroidal current, flowing through the supports connecting to the vessel. Conversely, the divertor is very highly segmented, which means that the contribution of the current density pattern (shown in Fig. 5) to passive stabilization is expected to be weaker.

**Figure 6:**
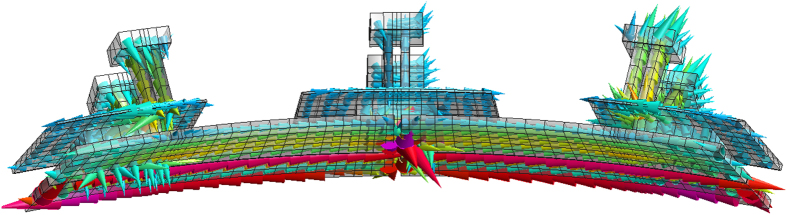
Current density distribution in passive plate.

The situation in the passive plates is more complex; the corresponding current density pattern is reported in Fig. 6. The supports are of two types: “indepedent supports” (the two lateral ones in Fig. 6) and “common supports” (the central one in Fig. 6). The common supports connect two toroidally consecutive passive plates, bridging the toroidal gap, while the independent supports do no. Both the supports also locally bridge the poloidal gap running in the toroidal direction. Both supports feature a “support plate” behind the passive plate, in which some current loops are induced. The net current flowing in the common support is zero; its overall effect is only to bridge the toroidal gap. In the end, the current path shown in Fig. 6 is rather complex. There are some current loops both in the passive plate and in the support plates located behind, superimposed to a net toroidal current running as follows: from the vessel to the plate through the independent support; along the plate in the toroidal direction; from one plate to the consecutive one through the common support bridging the gap; along the plate in the toroidal direction; from the plate back to the vessel through the following independent support. In the end, the actual toroidal span of the net toroidal current in the passive plates is twice as large as in the other PFCs, thanks to the presence of common supports. This enhances the contribution of passive plates to passive stabilization.

To check these conjectures, we computed the growth rate and the stability margin for two shots of the database, fictitiously eliminating one of the conducting structures at a time. This way, we can evaluate the actual contribution of various structures to passive stabilization. Table 3 reports the results: the first column corresponds to the reference case; cases A,B,C,D,E are obtained eliminating respectively: the passive plates, the low field plates, the high field plates, the upper divertor, the lower divertor. These results fully confirm the expectations reported above.

A deeper insight of the situation can be gained by deriving an equivalent axisymmetric model, with the same procedure applied to the old geometry of EAST^13^. Specifically, a fictitious toroidally continuous axisymmetric plasma facing component structure has been considered, but displaced towards the vessel as compared to the true location. The equivalent position and resistance of such structures are chosen so as to fit the growth rate and the stability margin of the 3D model^13^.

In particular, to get a good agreement (see Table 1) the PFCs are displaced of 4.4 cm towards the vessel and the resistivity is amplified of a factor 1.21. This confirms that in fact the stabilizing 3D current density pattern shown in Figs 4, 5 and 6 has a barycenter which is intermediate between the PFCs and the vessel due to its peculiar “zig-zag” behaviour, running partly in the vessel and partly in the PFCs. The increase of the resistivity takes into account the additional resistance of the supports.

## Conclusion

We have presented clear evidence of passive stabilization in EAST provided by toroidally segmented PFCs, in situations in which the toroidally continuous vessel is too far from plasma to give a contribution in this respect. A deep numerical analysis has demonstrated that this effect requires the presence of supports connecting PFCs to the vessel so as to give rise to intrinsically 3D “zig-zag” current density patterns with a net toroidal current flowing partly in the vessel and partly in the PFCs.

These results demonstrate that it is possible to conceive a similar stabilization mechanism also in future fusion devices, including power plants, in which the vessel will be very far from the plasma due to the need of thick blankets. Moreover, the availability of a computational tool, able to accurately describe this situation and predict the 3D stabilization effect, can provide a significant support to the design.

## Additional Information

**How to cite this article**: Chen, S. L. *et al.* 3D passive stabilization of *n* = 0 MHD modes in EAST tokamak. *Sci. Rep.* **6**, 32440; doi: 10.1038/srep32440 (2016).

## Figures and Tables

**Table 1:** Growth rates in *s* ^−1^.

Shot	experimental	3D model	2D model	vessel only	no supports	equivalent 2D
51406	103	101	72	1293	199	109
52688	80	79	61	622	139	90
52691	81	82	65	815	151	96
52692	76	76	59	573	134	86
52718	121	116	85	N.A.	243	132
54004	314	295	157	N.A.	2442	298
54006	305	298	158	N.A.	2557	302
54007	279	262	144	N.A.	1545	264
56059	157	130	91	N.A.	302	144
56773	275	296	146	N.A.	2531	269
56775	278	318	152	N.A.	3301	285
56848	208	214	125	N.A.	923	217
56849	205	214	125	N.A.	915	216
56871	206	213	124	N.A.	912	216
57362	159	148	99	N.A.	385	162
57364	161	150	101	N.A.	391	164
57393	159	140	94	N.A.	347	151

**Table 2:** Stability margins.

Shot	3D model	2D model	equivalent 2D
51406	0.83	1.13	0.83
52688	1.01	1.34	1.00
52691	0.96	1.29	0.95
52692	1.04	1.38	1.04
52718	0.74	1.02	0.72
54004	0.38	0.60	0.37
54006	0.38	0.60	0.37
54007	0.42	0.64	0.41
56059	0.68	0.95	0.67
56773	0.38	0.61	0.39
56775	0.36	0.59	0.37
56848	0.47	0.72	0.47
56849	0.47	0.72	0.48
56871	0.47	0.72	0.48
57362	0.63	0.90	0.62
57364	0.62	0.89	0.61
57393	0.66	0.93	0.67

**Table 3:** Growth rates *γ* and stability margins *m*_*s*_ for different assumptions.

Shot	Reference		Case A no passive plates		Case B no low field plates		Case C no high field plates		Case D no upper divertor		Case E no lower divertor	
	*γ*[*s*^−1^]	*m*_*s*_	*γ*[*s*^−1^]	*m*_*s*_	*γ*[*s*^−1^]	*m*_*s*_	*γ*[*s*^−1^]	*m*_*s*_	*γ*[*s*^−1^]	*m*_*s*_	*γ*[*s*^−1^]	*m*_*s*_
51406	101	0.83	138	0.62	112	0.78	132	0.73	106	0.76	115	0.77
56871	213	0.47	359	0.30	247	0.43	295	0.39	230	0.42	258	0.42
